# Lupus anticoagulant-hypoprothrombinemia syndrome with severe bleeding diathesis after coronavirus disease 2019: a case report

**DOI:** 10.3325/cmj.2022.63.490

**Published:** 2022-10

**Authors:** Tomislav Brblić, Klara Brčić, Sandra Margetić, Dubravka Čaržavec, Petar Gaćina, Nikolina Bogdanić

**Affiliations:** 1 Department of Hematology, Sestre Milosrdnice University Hospital Center, Zagreb, Croatia; 2 Department of Clinical Chemistry, Sestre Milosrdnice University Hospital Center, Zagreb, Croatia; 3 Dr. Fran Mihaljević University Hospital for Infectious Diseases, Zagreb, Croatia

## Abstract

Acquired antibodies against factor II (prothrombin) are rare and most commonly associated with severe liver disease or vitamin K antagonist treatment. In very rare cases, these antibodies and associated hypoprothrombinemia are found in patients with lupus anticoagulant (LAC), an antiphospholipid antibody that inhibits phospholipid-dependent coagulation tests. This uncommon entity, called lupus anticoagulant-hypoprothrombinemia syndrome (LAHPS), may cause both severe, life-threatening bleeding and a predisposition to thrombosis. Coronavirus disease 2019 (COVID-19) is associated with a variety of coagulation abnormalities and an increased risk of thrombosis. Bleeding may occur, but it is less common than thromboembolism and has mostly been described in association with the severity of the disease and anticoagulation treatment in hospitalized patients, rarely in the post-acute phase of the disease. We report on a case of an 80-year-old man who developed LAHPS with prothrombin antibodies and severe bleeding after COVID-19.

Coronavirus disease 2019 (COVID-19) is associated with hypercoagulable state and an increased risk of thromboembolism, both during acute infection and in post-COVID period (1). Most common coagulation abnormalities in COVID-19 patients are elevated D-dimer levels and fibrin split products (2). Other coagulation abnormalities, such as low fibrinogen levels, prolonged prothrombin time, and low platelet count, have also been reported (3). Despite all these derangements in coagulation tests, abnormal bleeding is uncommon.

## CASE REPORT

We report on a case of an 80-year-old man whose medical history included arterial hypertension, permanent atrial flutter, chronic kidney disease, benign prostatic hyperplasia, and amputated right lower leg due to arterial thrombosis. His medications included daily ramipril and warfarin (dose between 0.75-1.5 mg/d) with stable prothrombin time (PT) values within the therapeutic range of international normalized ratio (INR; 2-3.5) since May 2020. His symptoms started on May 1, 2021 with fever up to 38 °C (axillary), fatigue, and dry cough. He was not vaccinated against COVID-19. He was admitted to the hospital for Infectious Diseases in Zagreb, Croatia, on May 14 due to COVID-19 pneumonia, lower oxygen saturation (93% on room air), and comorbidity burden. A chest radiograph at admission showed bilateral interstitial lesions. Laboratory tests at the admission showed warfarin overdose (INR>6.2, PT activity <6%), without signs of bleeding, which was treated with 10 mg of vitamin K intravenously without adequate response, and 2x250 ml of fresh frozen plasma (FFP) was administered at the physician’s discretion. Coagulation tests normalized (INR 1.6, PT activity 41%), and enoxaparin was started two days after admission. As the patient’s oxygen saturation at the admission was 93% on room air and since he was mildly tachypneic (respiratory rate 26/min), he was also treated with dexamethasone intravenously (6 mg/d) for 11 days, and co-amoxiclav intravenously for urinary tract infection (*Enterococcus faecalis, Escherichia coli*). Three days before discharge, warfarin was again started until the therapeutic value of PT was established (INR 2.2, PT activity 30% when discharged). During his hospital stay, he did not require oxygen supplementation and was afebrile. He was discharged to home care on May 24 while eupneic, with normal oxygen saturation and without any signs of bleeding, with a recommendation of using dexamethasone 4 mg/d for the next three days (total of 14 days).

On May 29, the patient presented at the emergency department of a university hospital with shortness of breath and dysuria. A chest CT scan showed diffuse post-COVID fibrotic changes with residual inflammatory infiltrates. Coagulation tests were within the therapeutic range (INR 3.5, PT activity 20%). He was discharged with a recommendation to take cefpodoxime for 10 days due to urinary tract infection. On June 25, he was examined again at the emergency department due to rectal bleeding. PT testing showed INR value above the therapeutic range (INR 4.3, PT activity 13%). There was no need for erythrocyte concentrate transfusion (hemoglobin level 10.4 g/dL). He was discharged with a recommendation of warfarin dose reduction (no bleeding was observed during the assessment).

**Figure 1:**
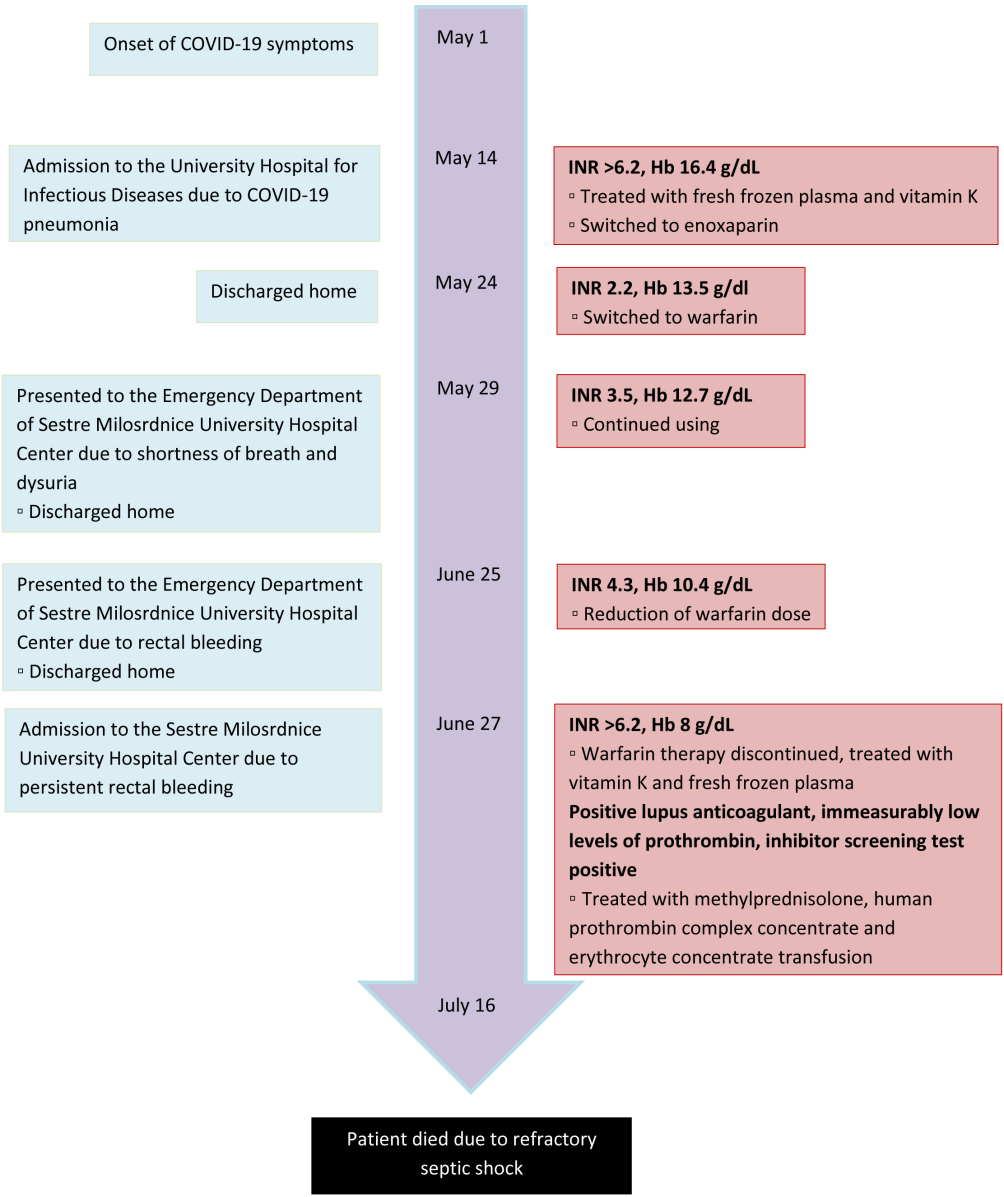
The most important events and laboratory findings. COVID-19 – coronavirus disease 2019; INR – international normalized ratio; Hb – hemoglobin.

On June 27, the patient was admitted to a university hospital due to persistent rectal bleeding. Upon admission, his laboratory tests showed INR>6.2 and immeasurably low PT activity (<6%), with a significant drop in hemoglobin level (8.0 g/dL). Warfarin therapy was discontinued. Upper and lower gastrointestinal tract endoscopy was performed, with no active bleeding site visualized, but intact intestinal mucosa was bleeding upon minor contact. Although the patient was receiving intravenous vitamin K (10 mg) and FFP (250-500 mL) daily, PT activity remained immeasurably low (<6%). Additional tests showed positive LAC, with LAC ratio 1.99 (reference value <1.37) and immeasurably low levels of factor II (FII, prothrombin) (<5% of activity, reference range 70%-120%). Activities of other coagulation factors were within the reference ranges (FV, FVII, FX, FXI, FXII, FXIII) or marginally above (FI, FVIII). Inhibitor screening for prothrombin antibodies was positive including mixing test with normal plasma, with residual activity of test mixture with control mixture of 42%. These results indicated the presence of LAHPS. Serology tests for the detection of underlying autoimmune diseases were all negative (anticardiolipin antibodies IgM, IgG, anti-β2 glycoprotein I antibodies IgM, IgG, antinuclear antibodies, anti-dsDNA antibodies). Abdominal ultrasound showed no signs of spleen/liver enlargement or intraabdominal lymphadenopathy. Methylprednisolone was started on the eighth day of hospital stay, 80 mg daily (1 mg/kg) during 11 days. Due to persistent bleeding (insufficient rise in hemoglobin levels despite red blood cells transfusion, continuous melena), human prothrombin complex concentrate (Octaplex, Octapharma, Vienna, Austria) was also administered (2000 IJ for the first dose, additional 2x1000 IJ applied when bleeding was observed again). Laboratory tests showed only partial correction of PT and FII (maximum PT activity 37%, INR 1.9; maximum FII activity level 32%) and stabilization of hemoglobin level (lowest 5.8 g/dL on the fourth and eleventh day of hospital stay; later, when bleeding stopped, between 9.0 and 10.6 g/dL). The case timeline is shown in Figure 1, and laboratory values are shown in Table 1. Unfortunately, the patient died on the 19th day of hospital stay due to clinical and laboratory signs of nosocomial urosepsis (*Klebsiella oxytoca* ESBL isolated from his urine and blood culture) and refractory shock.

**Table 1:** The most important laboratory findings, May-July 2021

	Reference range	May			June			July				
Date		14	24	29	25	27	30	4	7	9	12	15
Hemoglobin, g/dL	13.8-17.5	16.4	13.5	12.7	10.4	8.0	5.8	8.3	5.8	9.3	10.3	9.0
Prothrombin time, %	≥70*	<6	29	19	13	<6	<6	<6	22	26	25	37
International normalized ratio	≤1^†^	>6.2	2.2	3.5	4.3	>6.2	>6.2	>6.2	2.8	2.4	2.6	1.9
Factor II, %	70-120							<5	20	32	28	28
Lupus anticoagulant ratio	<1.37							1.99				

*therapeutic range 20%-35%.

†therapeutic range 2-3.5.

## DISCUSSION

Acquired inhibitors of coagulation causing bleeding manifestations are rare and usually associated with autoimmune disease, drug ingestion, or in response to replacement therapy in patients with hereditary coagulation factor deficiencies (4). Acquired factor-II antibodies are most commonly associated with antiphospholipid syndrome (APS) (5). Most of APS patients do not have hypoprothrombinemia; if they do, bleeding is still uncommon because of a counterbalancing prothrombotic effect of LAC (5). Several studies found an increased incidence of LAC and other antiphospholipid antibodies in COVID-19 patients, with no confirmed association with either thrombosis or bleeding (6-8). These antibodies are sometimes found after other viral infections, and are usually transient, without thrombotic/bleeding complications (8). LAHPS is a rare entity reported in association with only a few conditions, such as primary APS, systemic lupus erythematosus, viral infections, and occasionally drug administration and lymphoma (9,10). Patients with LAHPS can have severe bleeding complications, but may also have thrombotic events (9,10). Both incidences are more common in patients with underlying autoimmune diseases and lymphomas than in patients who develop LAHPS after infection (9,10). Treatment of LAHPS presents a specific challenge, as it requires maintaining an optimal balance between bleeding and thrombosis (9). Corticosteroids at the dose of 1 mg/kg are considered the first-line treatment, which in most cases leads to normalization of both PT and factor II levels (10). The effectiveness of other therapeutic options (azathioprine, cyclophosphamide, rituximab, intravenous immunoglobulins) is unclear as they were administered only to a few patients, usually in combination with corticosteroids (9,10). The impact of supportive treatment (vitamin K, FFP, platelet concentrates, prothrombin complex concentrates, recombinant factor VII, packed red blood cells) is also difficult to evaluate because of its coadministration with corticosteroids or other immunosuppressants (10). Other treatment-related complications are serious secondary infections due to immunosuppression (9,10), as was the case in our patient. To our knowledge, this is the first case of LAHPS with severe bleeding diathesis strongly associated with previous COVID-19 infection. This case adds to the complexity of COVID-19-related coagulopathy, which includes immune, endothelial, and coagulation systems, and calls attention to the possibility of bleeding complications, especially in patients using therapeutic anticoagulants (1-3).
